# Triazole phenotypes and genotypic characterization of clinical *Aspergillus fumigatus* isolates in China

**DOI:** 10.1038/emi.2017.97

**Published:** 2017-12-06

**Authors:** Shuwen Deng, Lili Zhang, Yanfeng Ji, Paul E Verweij, Kin Ming Tsui, Ferry Hagen, Jos Houbraken, Jacque F Meis, Parida Abliz, Xiaodong Wang, Jingjun Zhao, Wanqing Liao

**Affiliations:** 1 Department of Medical Microbiology, People’s Hospital of Suzhou National New & Hi-Tech Industrial Development Zone, Jiangsu 215219, China; 2 Department of Dermatology, Tongji Hospital of Tongji University, Tongji University School of Medicine, Shanghai 200065, China; 3 Department of Medical Microbiology, Radboud University Medical Centre, Nijmegen 6500HB, The Netherlands; 4 Division of Infectious Diseases, Faculty of Medicine, University of British Columbia, Vancouver V6H3Z6, Canada; 5 Department of Medical Microbiology & Infectious Diseases, Canisius Wilhelmina Hospital, Nijmegen 6500GS, The Netherlands; 6 Centre of Expertise in Mycology Radboud University Medical Centre/CWZ, Nijmegen 6500HB, The Netherlands; 7 Westerdijk Fungal Biodiversity Institute, Utrecht 3584CT, The Netherlands; 8 First Hospital of Xinjiang Medical University, Urumqi 830054, China; 9 Shanghai Key Laboratory of Molecular Medical Mycology, Changzheng Hospital, Second Military Medical University, Shanghai 200003, China

**Keywords:** antifungal susceptibility testing, drug resistance, drug target mutation, epidemiology, microsatellite markers, triazole-resistant

## Abstract

This study investigated the triazole phenotype and genotypic of clinical *Aspergillus fumigatus* isolates from China. We determined the triazole susceptibility profiles of 159 *A. fumigatus* isolates collected between 2011 and 2015 from four different areas in China tested against 10 antifungal drugs using the Clinical Laboratory Standard Institute M38-A2 method. For the seven itraconazole-resistant *A. fumigatus* isolates identified in the study, the *cyp51A* gene, including its promoter region, was sequenced and the mutation patterns were characterized. The resistant isolates were genotyped by microsatellite typing to determine the genetic relatedness to isolates from China and other countries. The frequency of itraconazole resistance in *A. fumigatus* isolates in our study was 4.4% (7/159). Six of the seven triazole-resistant isolates were recovered from the east and southeast of China, and one from was recovered from the west of China. No resistant isolates were found in the north. Three triazole-resistant isolates exhibited the TR_34_/L98H mutation, two carried the TR_34_/L98H/S297T/F495I mutation and one harbored a G54V mutation in the *cyp51A* gene. Analysis of the microsatellite markers from seven non-wild-type isolates indicated the presence of five unique genotypes, which clustered into two major genetic groups. The *cyp51A* gene mutations TR_34_/L98H and TR_34_/L98H/S297T were the most frequently found mutations, and the G54V mutation was reported for the first time in China. The geographic origin of the triazole-resistant isolates appeared to concentrate in eastern and south-eastern areas, which suggests that routine antifungal susceptibility testing in these areas should be performed for all clinically relevant *A. fumigatus* isolates to guide antifungal therapy and for epidemiological purposes.

## INTRODUCTION

Invasive aspergillosis (IA) in immunocompromised patients results in substantial morbidity and mortality.^1, 2^ More than 40 *Aspergillus* species have been reported as causal agents of IA, and *Aspergillus fumigatus* is the leading pathogen in humans in most regions of the world.^2, 3^ Antifungal agents such as the triazoles (itraconazole, posaconazole, voriconazole), the polyenes (e.g., amphotericin B) and the echinocandins are commonly prescribed drugs for patients diagnosed with IA.^4, 5^ Recently, the antifungal azole isavuconazole was licensed for primary therapy for IA.^6^ The key to successful treatment of IA includes early and accurate diagnosis and appropriate antifungal therapy at an adequate dosage. However, rapid, accurate and sensitive diagnosis is often a challenge in clinical laboratories,^7^ and antifungal therapy is further complicated by the emergence of triazole resistance in *A. fumigatus*.^8, 9^ It has been suggested that triazole resistance among *Aspergillus* species is more common than currently recognized.^9^ Recently, an expert panel recommended that initial treatment regimens for IA should take into account the local drug resistance frequency of *A. fumigatus*.^10^ Although triazole resistance has been reported in Asia,^11, 12, 13, 14^ only a few Chinese surveillance reports on the antifungal susceptibility of clinical *A. fumigatus* isolates are available. Most reports come from restricted geographic areas and consider only a modest number of isolates or relatively few antifungal agents.^14, 15, 16, 17, 18^ Given the lack of comprehensive information on the triazole resistance of isolates causing aspergillosis in China, the objectives of this study were to investigate the following: (1) the susceptibility of clinical *A. fumigatus* isolates from different areas in China to 10 antifungal drugs; (2) the triazole phenotypes and the mutation patterns in the *cyp51A* gene of resistant isolates; and (3) the genotypic relationships among azole-resistant isolates using microsatellite typing.^19^

## MATERIALS AND METHODS

### Isolates

A total of 159 clinical isolates, including 37 from eastern areas, 39 from the south-eastern areas, 61 from northern areas and 22 from western areas, were collected between 2011 and 2015 in various medical centers from different geographic areas of China. Ethical approval was obtained, and all patients involved understood and agreed to the usage of these clinical specimens in the present study. All isolates were identified to the species level by sequencing the partial β-tubulin gene (*benA*) as described previously.^16^ The obtained sequences were compared with the NCBI nucleotide database and the internal sequence database of the Westerdijk Fungal Biodiversity Institute containing verified *benA* sequences of *Aspergillus* section *Fumigati*. The geographical origin, clinical data and GenBank accession numbers for the generated *benA* sequences are listed in Supplementary Table S1.

### Antifungal susceptibility testing

All isolates were tested for antifungal susceptibility under conditions described in the Clinical Laboratory Standard Institute M38-A2 reference method.^20^ The antifungals amphotericin B, caspofungin, itraconazole, posaconazole, terbinafine and voriconazole were obtained from Sigma-Aldrich (Basingstoke, UK), and anidulafungin, micafungin, isavuconazole and ravuconazole were obtained from Toronto Research Chemicals Inc. (Toronto, Canada). The tested concentrations ranged from 0.008 to 4 mg/L for the echinocandins (anidulafungin, caspofungin and micafungin) and from 0.031 to 16 mg/L for the other compounds. All isolates were cultured on potato dextrose agar at 35 °C for 3–5 days and subcultured at least twice to ensure viability and purity. Conidia were harvested using sterile saline with Tween 20, and the final inoculum concentration of the suspension was adjusted to 0.4–5 × 10^4^ colony-forming units (CFU)/mL in RPMI 1640 buffered with morpholinepropanesulfonic acid. Plates were incubated for 48 hours at 35 °C.^20^ Both minimum inhibitory concentrations (MIC) and minimum effective concentrations (MEC) were determined microscopically (Primo Star Zeiss, Jena, Germany) at × 40 magnification. Epidemiological cutoff values (ECVs) were used to classify triazole susceptibility and to detect non-wild-type isolates.^20, 21, 22^ Isolates were considered wild type when the MIC was equal to or lower than the ECV and non-wild type when the MIC was higher than the ECV. Isolates with MIC values >2 mg/L for amphotericin B,^23^ >1 mg/L for isavuconazole, itraconazole and voriconazole and MIC values >0.5 mg/L for posaconazole were considered non-wild type (potentially resistant or less susceptible isolates).^24^ There are no ECVs currently available for the echinocandins, ravuconazole or terbinafine. Quality control was performed as recommended in Clinical Laboratory Standard Institute document M38-A2 using strains *A. fumigatus* ATCC MYA-3627 and *C. parapsilosis* ATCC 22019.^25^ All experiments for each isolate were performed using three independent replicates on different days.

### Sequencing of A. fumigatus cyp51A gene

Non-wild-type *A. fumigatus* isolates were selected for detection of *cyp51*A mutations. Genomic DNA was extracted, and the full sequences of the *cyp51*A gene with the promoter region were amplified and sequenced (the primers used are listed in Supplementary Table S2).^26^ The sequences obtained were aligned with the sequence from a triazole-susceptible isolate (GenBank accession AF338659) using ClustalW.^27^ After the removal of the non-coding intron region, the predicted *cyp51*A amino-acid sequence was screened for substitutions, particularly those linked to triazole resistance.

### Microsatellite genotyping

Microsatellite typing was used to determine the genetic relationships among the triazole-resistant *A. fumigatus* isolates. Nine loci were amplified in three multiplex-PCR assays, and subsequent fragment analysis was performed using the methods described previously.^28^ Data were analyzed using Bionumericsv7.5 (Applied Maths, Sint-Martens-Latem, Belgium), and the dendrogram was generated using the categorical similarity coefficient followed by UPGMA cluster analysis implemented in Bionumerics. Additional microsatellite data from 18 clinical *A. fumigatus* isolates from China and 14 isolates from other countries such as Australia, Netherlands, India, Japan and Germany were included to provide additional insight into the genetic relationships among the triazole-resistant isolates.^13, 14, 29, 30, 31, 32^

### Statistical analysis

The geometric means, MIC/MEC, modal MIC/MEC, MIC /MEC ranges and MIC_90_ (MIC/MEC at which 90% of the isolates tested were inhibited) were measured for all isolates. Kruskal–Wallis testing was performed to test for significant differences between the MIC/MEC for each drug among four geographical areas using SPSS package v 20.0 (IBM Corp., Armonk, NY, USA). The differences were considered statistically significant at a *P*-value≤0.05 (two-tailed).

## RESULTS

The MIC/MEC ranges, modal MIC/MEC, distribution of MICs/MECs of the 10 antifungal agents and the percentage of triazole-resistant isolates among the 159 isolates of *A. fumigatus* are presented in Table 1. Anidulafungin and micafungin were the most active drugs against *A. fumigatus in vitro* as they had the lowest modal MICs/MECs (mg/L) (0.016 (*n*=61) and 0.031 (*n*=64), respectively), followed by posaconazole (0.125 (*n*=72)), caspofungin (0.25 (*n*=119)), ravuconazole (0.25 (*n*=112)), voriconazole (0.25 (*n*=103)), itraconazole (0.5 (*n*=93)), amphotericin B (1 (*n*=119)), isavuconazole (1 (*n*=88)) and terbinafine (2 (*n*=79)).

The MIC values of the triazoles (except voriconazole) varied significantly among the four geographic areas (Table 2). The activity of itraconazole against western isolates was the most potent, whereas eastern isolates were less susceptible. In contrast, for posaconazole and ravuconazole, most *A. fumigatus* isolates from the western area had higher GM MICs than isolates in the other three areas; for isavuconazole, isolates from the east and the southeast had higher MICs than isolates from the north and the west. However, all isolates of *A. fumigatus* were particularly susceptible to the three echinocandins, although isolates from the west had lower MECs compared with isolates from the other areas (Table 2).

Seven isolates with MIC values above the established ECV for isavuconazole, itraconazole, posaconazole and voriconazole were identified, and the corresponding mutations in the *cyp51A* gene region and their geographical origins are shown in Table 3.

The triazole-resistance rates for clinical isolates of *A. fumigatus* in the four geographic areas were variable, with 10.8% in the east, 5.1% in the southeast, 4.5% in the west and 0% in the north.

**Figure 1:**
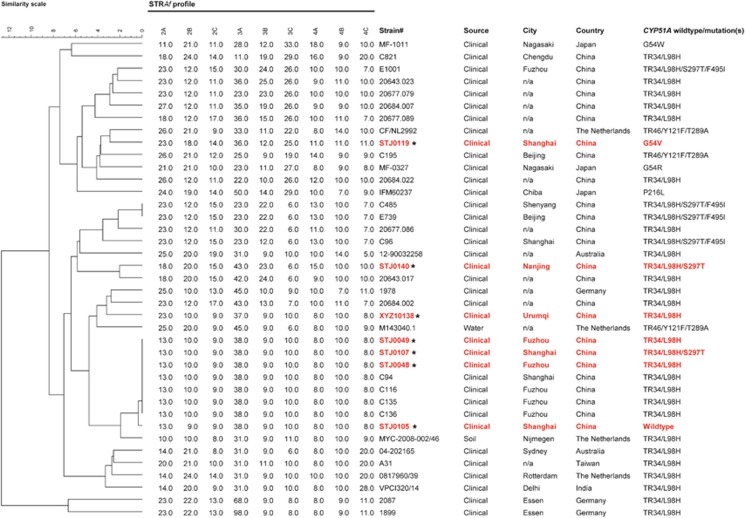
Genotypic analysis of triazole-resistant *Aspergillus fumigatus* clinical isolates, including seven triazole-resistant isolates in this study, and analyses published previously from China and other countries. The dendrogram is based on a categorical analysis of nine microsatellite markers in combination with the unweighted Pair Group Method with arithmetic mean clustering. The scale bar indicates the percentage identity. *Denotes the seven clinical Chinese isolates in this study.

Analysis of microsatellite markers of the seven itraconazole-resistant isolates indicated the presence of five unique genotypes that clustered into two major independent genetic groups (Figure 1). The genetic profiles of isolates STJ0119, STJ0140 and XYZ10138 were unique and were different from other isolates in the tree. They were distantly related to many Chinese isolates reported in previous studies.^14, 16^ Three isolates (STJ0049, STJ0107 and STJ0048) were identical in their microsatellite profiles, and they were also genetically identical to four clinical isolates from China from previous studies (Figure 1). These seven isolates appeared to be highly clonal based on the microsatellites.

## DISCUSSION

Our study showed that clinical *A. fumigatus* isolates from different areas in China have variable susceptibility profiles toward 10 common antifungal drugs, including two novel triazole antifungal agents: isavuconazole and ravuconazole. Despite the variability in drug susceptibilities, anidulafungin and micafungin were the most active/effective drugs (Table 1), and triazoles were active against >95.6% (*n*=152/159) of the isolates, which is in agreement with other studies.^33, 34, 35^ The novel triazoles isavuconazole and ravuconazole also had good *in vitro* activity against *A. fumigatus* (96.2% inhibition at MIC≤1 mg/L (*n*=153/159)). The *in vitro* activity of isavuconazole against *A. fumigatus* (modal MIC 1 mg/L) was similar to the activity of itraconazole (modal MIC 0.5 mg/L) but lower than either posaconazole (modal MIC 0.125 mg/L) or ravuconazole (modal MIC 0.25 mg/L) and voriconazole (modal MIC 0.25 mg/L), which was comparable to previous reports.^6, 33, 34, 35, 36^ Nevertheless, ravuconazole showed excellent activity against *A. fumigatus*, as previously reported.^36, 37^

In a 5-year period, the rate of triazole resistance in *A. fumigatus* isolates in our study was 4.4% (*n*=7/159), and this percentage was similar to the current global prevalence of triazole resistance in *Aspergillus* (3–6%).^10^

Five of the seven resistant isolates exhibited a TR_34_/L98H or TR_34_/L98H/S297T mutation in the *cyp51A* gene, confirming the presence of TR_34_/L98H mutations in China.^14, 15, 16^ The TR_34_/L98H mutation has been associated with exposure to azole fungicides in the environment rather than triazole therapy in patients.^38^ Strikingly, seven such isolates in China (three from current study) showed no genetic variability, albeit with two different mutation patterns, suggesting a possible single and recent origin for these resistant isolates.

Variability in resistance frequency was observed in our study: triazole-resistant *A. fumigatus* was concentrated in the east (four non-wild-type isolates) and southeast (two non-wild-type isolates). One triazole-resistant isolate was obtained from the western area (Table 2), thousands of kilometers distant from the east. A similar variation in triazole-resistance prevalence between centers was found in the Netherlands.^7^ Differences in resistance frequencies between medical centers might reflect differences in environmental exposure to triazole-resistant *A. fumigatus*. Further studies are needed to identify local environmental niches as they are probably critical to decrease the exposure of patients to *A. fumigatus* harboring these resistance mutations. Azole resistance in *A. fumigatus* due to non-cyp51A mechanisms is also increasingly reported,^39^ which including activation of efflux pumps, in particular the overexpression of adenosine triphosphate-binding cassette transporters, transporters of the major facilitator superfamily, transcription factors, and non-synonymous mutations. The mutation P88L in HapE, an important subunit of the CCAAT-binding transcription factor complex, was found to confer resistance in *A. fumigatus*.^40^ The occurrence of genomic deletions and non-synonymous mutations in genes (*afyap1* and *aldA*) other than *cyp51A* has been described in *A. fumigatus* as possibly leading to azole resistance.^41^

*A. fumigatus* isolates harboring the mutation TR_34_/L98H are found globally, and in this study, they conferred high MICs to all five triazole drugs. The results were different with the TR_34_/L98H/S297T mutants, which had lower voriconazole MICs (Table 3). This discrepancy had previously been noted, and we suggested at that time that the extra S297T mutation might represent a compensatory mutation.^42^ The strain with the G54 point mutation represents the first report from China. Recently, this mutation in *cyp51A*, known previously from Europe and India, was also reported in Argentina.^39, 43, 44^ Two new azoles, ravuconazole and isavuconazole, which are not yet approved for clinical use in China, showed reduced *in vitro* activity against itraconazole-resistant *A. fumigatus* isolates. This result is probably due to azole cross-resistance: 85.7% (*n*=6/7) of the itraconazole-resistant isolates were also resistant to ravuconazole and isavuconazole, and 71.4% (*n*=5/7) were resistant to posaconazole (Table 3). The isolate (STJ0119) with the G54 mutation was only resistant to itraconazole (MIC>16 mg/L) but not to the other triazoles.^45^ This isolate was obtained from a patient admitted to a hospital in Shanghai with azole preexposure in the period before isolation. Unfortunately, we have no detailed information regarding the use of azole drugs in this patient. However, the TR_46_/Y121F/T289A combination of mutations was not found in this study, although recently a clinical isolate was reported from Beijing, China.^15^

The geographical variation in *A. fumigatus* azole-resistant isolates suggests a need to include local drug resistance rates to devise public health policies and local guidelines for treatment and management. Furthermore, in the east and the southeast, where resistant isolates are prevalent, routine antifungal susceptibility testing should be performed for all clinically relevant *A. fumigatus* isolates to guide antifungal therapy and for epidemiological purposes.

## Figures and Tables

**Table 1:** MIC/MEC ranges, modal of MICs/MECs, distribution of MICs/MECs (mg/L) obtained by testing the susceptibility of 159 *A. fumigatus* isolates to 10 antifungal agents and the percentage of non-WT isolates for the 159 isolates of *A. fumigatus*

**Antifungal agent**	**MIC/MEC range**	**No. of isolates with MIC/MEC of**												**% of non-WT isolates**
Triazoles		0.008	0.016	0.031	0.063	0.125	0.25	0.5	1	2	4	8	16	
Itraconazole	0.063–>16				1		9	**93**	49				7	4.40
Voriconazole	0.063–2				1	17	**103**	24	13	1				0.63
Posaconazole	0.031–1			4	63	**72**	11	4	5					3.14
Isavuconazole	0.063–4				1	2	5	56	**88**	2	4			3.77
Ravuconazole	0.063–8				3	20	**112**	15	2	3	2	1		3.77
Echinocandins														
Micafungin	≤0.008–0.5	19	**61**	58	19	1		1						0
Anidulafungin	≤0.008–0.063	5	52	**64**	38									0
Caspofungin	0.125–0.5					10	**119**	30						0
Polyenes														
Amphotericin B	0.5–2							5	**119**	35				0
Allylamines														
Terbinafine	0.25–4						1	1	12	**79**	66			Unknown

Abbreviations: minimum inhibitory concentration, MIC; minimum effective concentration, MEC; values in bold indicate modal or most frequent MICs, Modal MIC/MEC; wild type WT. MICs are shown for amphotericin B, itraconazole, posaconazole, voriconazole, ravuconazole, isavuconazole; MECs are shown for micafungin, caspofungin and anidulafungin.

**Table 2:** Comparisons of activities of eight antifungal drugs tested against *A. fumigatus* isolates in four geographic areas

**Antifungal agents**	**Geometric mean (MIC**_**90**_**/MEC**_**90**_**) (mg/L) for isolates from:**			
	**East (*****n*****=37)**	**South-east (*****n*****=39)**	**North (*****n*****=61)**	**West (*****n*****=22)**
Itraconazole	0.894 (16)^#^	0.752 (1)	0.658 (1)^#^	0.485 (1)*
Voriconazole	0.290 (0.5)	0.273 (1)	0.296 (0.5)	0.302 (0.5)
Posaconazole	0.116 (0.5)	0.113 (0.25)	0.091 (0.125)^#^	0.137(0.25)*
Ravuconazole	0.290 (2)^#^	0.264 (0.25)^#^	0.228 (0.25)^#^	0.401 (0.5)*
Isavuconazole	0.894 (2)^#^	0.915 (1)^#^	0.672 (1)*	0.624 (1)
Micafungin	0.029 (0.063)^#^	0.023 (0.063)^#^	0.022 (0.031)^#^	0.014 (0.015)*
Anidulafungin	0.030 (0.063)^#^	0.035 (0.063)^#^	0.028 (0.063)^#^	0.016 (0.015)*
Caspofungin	0.319 (0.5)^#^	0.264 (0.25)^#^	0.287 (0.5)^#^	0.194 (0.25)*

Abbreviations: minimum effective concentration, MEC; minimum inhibitory concentration, MIC. Note: The one with * means that it had statistical difference (*P*<0.01) when compared with the one with #.

**Table 3:** MICs/MECs of seven triazole-resistant *A. fumigatus* isolates and their corresponding mutation type in the *cyp51A* gene region and geographical origin

**Isolates**	**MICs/MECs (mg/L)**										**Mutation type in** ***cyp51A*** **gene**	**Geographical origin**
	**Amb**	**Itr**	**Vor**	**Pos**	**Isa**	**Rav**	**Anid**	**Mic**	**Cas**	**Ter**		
STJ0048	1	>16	1	1	4	2	0.015	0.03	0.25	2	TR34/L98H	South-eastern area
STJ0049	1	>16	1	1	4	2	0.03	≤0.008	0.25	2	TR34/L98H	South-eastern area
STJ0105	1	>16	1	1	4	2	0.06	0.03	0.25	2	—	Eastern area
STJ0107	1	>16	0.5	1	2	4	0.03	0.03	0.5	2	TR34/L98H/S297T	Eastern area
STJ0119	0.5	>16	0.125	0.5	1	0.125	0.03	0.03	0.5	1	G54V	Eastern area
STJ0140	0.5	>16	0.5	1	2	8	0.06	0.06	0.5	2	TR34/L98H/S297T	Eastern area
XJ138	1	16	2	0.5	4	4	0.015	0.015	0.125	2	TR34/L98H	Western area

Abbreviations: amphotericin B, Amb; anidulafungin, Anid; caspofungin, Cas; isavoconazole, Isa; itraconazole, Itr; minimum effective concentration, MEC; micafungin, Mic; minimum inhibitory concentration, MIC; posaconazole, Pos; ravuconazole, Rav; terbinafine, Ter; voriconazole, Vor.
