# Supplementary material for: Triazole phenotypes and genotypic characterization of clinical Aspergillus fumigatus isolates in China
Source: Emerg Microbes Infect. 2017 Dec 6;6(12):e109–. doi: 10.1038/emi.2017.97 (PMC5750463; doi:10.1038/emi.2017.97)
Supplement: Supplementary Table S1 [file emi201797x1.docx]

**Supplementary Table S1** Information of the159 *A. fumigatus* isolates tested in this study

| Strains No. | Species name | Source | AccessionNo.  (Genebank) | Geographical origin |
| --- | --- | --- | --- | --- |
| SHTJ0001 | *Aspergillus fumigatus* | bile | KT852582 | North(Beijing) |
| SHTJ0002 | *Aspergillus fumigatus* | pus | KT852583 | North(Beijing) |
| SHTJ0003 | *Aspergillus fumigatus* | sputum | KT852584 | North(Beijing) |
| SHTJ0004 | *Aspergillus fumigatus* | skin | KT852585 | North(Beijing) |
| SHTJ0005 | *Aspergillus fumigatus* | sputum | KT852586 | North(Beijing) |
| SHTJ0006 | *Aspergillus fumigatus* | sputum | KT852587 | North(Beijing) |
| SHTJ0007 | *Aspergillus fumigatus* | sputum | KT852588 | North(Beijing) |
| SHTJ0008 | *Aspergillus fumigatus* | sputum | KT852589 | North(Beijing) |
| SHTJ0009 | *Aspergillus fumigatus* | sputum | KT852590 | North(Beijing) |
| SHTJ0010 | *Aspergillus fumigatus* | sputum | KT852591 | North(Beijing) |
| SHTJ0011 | *Aspergillus fumigatus* | sputum | KT852592 | North(Beijing) |
| SHTJ0012 | *Aspergillus fumigatus* | sputum | KT852593 | North(Beijing) |
| SHTJ0013 | *Aspergillus fumigatus* | sputum | KT852594 | North(Beijing) |
| SHTJ0014 | *Aspergillus fumigatus* | sputum | KT852595 | North(Beijing) |
| SHTJ0015 | *Aspergillus fumigatus* | sputum | KT852596 | North(Beijing) |
| SHTJ0016 | *Aspergillus fumigatus* | paranasalsinus | KT852597 | North(Beijing) |
| SHTJ0017 | *Aspergillus fumigatus* | sputum | KT852598 | North(Beijing) |
| SHTJ0018 | *Aspergillus fumigatus* | sputum | KT852599 | North(Beijing) |
| SHTJ0019 | *Aspergillus fumigatus* | sputum | KT932336 | North(Beijing) |
| SHTJ0020 | *Aspergillus fumigatus* | paranasalsinus | KT852600 | North(Beijing) |
| SHTJ0021 | *Aspergillus fumigatus* | sputum | KT852601 | North(Beijing) |
| SHTJ0022 | *Aspergillus fumigatus* | sputum | KT852602 | North(Beijing) |
| SHTJ0023 | *Aspergillus fumigatus* | sputum | KT852603 | North(Beijing) |
| SHTJ0024 | *Aspergillus fumigatus* | sputum | KT852604 | North(Beijing) |
| SHTJ0025 | *Aspergillus fumigatus* | sputum | KT852605 | North(Beijing) |
| SHTJ0026 | *Aspergillus fumigatus* | sputum | KT852606 | North(Beijing) |
| SHTJ0027 | *Aspergillus fumigatus* | sputum | KT932337 | South(Fuzhou) |
| SHTJ0028 | *Aspergillus fumigatus* | sputum | KT852607 | South(Fuzhou) |
| SHTJ0029 | *Aspergillus fumigatus* | sputum | KT932338 | South(Fuzhou) |
| SHTJ0030 | *Aspergillus fumigatus* | sputum | KT852608 | South(Fuzhou) |
| SHTJ0031 | *Aspergillus fumigatus* | sputum | KT852609 | South(Fuzhou) |
| SHTJ0032 | *Aspergillus fumigatus* | sputum | KT852610 | South(Fuzhou) |
| SHTJ0033 | *Aspergillus fumigatus* | sputum | KT852611 | South(Fuzhou) |
| SHTJ0034 | *Aspergillus fumigatus* | throat swab | KT932339 | South(Fuzhou) |
| SHTJ0035 | *Aspergillus fumigatus* | -- | KT852612 | South(Fuzhou) |
| SHTJ0036 | *Aspergillus fumigatus* | sputum | KT932340 | South(Fuzhou) |
| SHTJ0037 | *Aspergillus fumigatus* | sputum | KT932341 | South(Fuzhou) |
| SHTJ0038 | *Aspergillus fumigatus* | -- | KT852613 | South(Fuzhou) |
| SHTJ0039 | *Aspergillus fumigatus* | sputum | KT852614 | South(Fuzhou) |
| SHTJ0040 | *Aspergillus fumigatus* | sputum | KT932342 | South(Fuzhou) |
| SHTJ0041 | *Aspergillus fumigatus* | sputum | KT932343 | South(Fuzhou) |
| SHTJ0042 | *Aspergillus fumigatus* | sputum | KT932344 | South(Fuzhou) |
| SHTJ0043 | *Aspergillus fumigatus* | sputum | KT932345 | South(Fuzhou) |
| SHTJ0044 | *Aspergillus fumigatus* | sputum | KT932346 | South(Fuzhou) |
| SHTJ0045 | *Aspergillus fumigatus* | sputum | KT932347 | South(Fuzhou) |
| SHTJ0046 | *Aspergillus fumigatus* | -- | KT852615 | South(Fuzhou) |
| SHTJ0047 | *Aspergillus fumigatus* | -- | KT932348 | South(Fuzhou) |
| SHTJ0048 | *Aspergillus fumigatus* | -- | KT932349 | South(Fuzhou) |
| SHTJ0049 | *Aspergillus fumigatus* | -- | KT852616 | South(Fuzhou) |
| SHTJ0050 | *Aspergillus fumigatus* | -- | KT932350 | South(Fuzhou) |
| SHTJ0051 | *Aspergillus fumigatus* | sputum | KT852617 | South(Fuzhou) |
| SHTJ0052 | *Aspergillus fumigatus* | sputum | KT852618 | South(Fuzhou) |
| SHTJ0053 | *Aspergillus fumigatus* | sputum | KT932351 | South(Fuzhou) |
| SHTJ0054 | *Aspergillus fumigatus* | -- | KT852619 | South(Fuzhou) |
| SHTJ0055 | *Aspergillus fumigatus* | sputum | KT932352 | South(Fuzhou) |
| SHTJ0056 | *Aspergillus fumigatus* | sputum | KT852620 | South(Fuzhou) |
| SHTJ0057 | *Aspergillus fumigatus* | sputum | KT852621 | South(Fuzhou) |
| SHTJ0058 | *Aspergillus fumigatus* | throat swab | KT932353 | South(Fuzhou) |
| SHTJ0059 | *Aspergillus fumigatus* | sputum | KT852622 | South(Fuzhou) |
| SHTJ0060 | *Aspergillus fumigatus* | -- | KT852623 | South(Fuzhou) |
| SHTJ0061 | *Aspergillus fumigatus* | -- | KT852624 | South(Fuzhou) |
| SHTJ0062 | *Aspergillus fumigatus* | -- | KT852625 | South(Fuzhou) |
| SHTJ0063 | *Aspergillus fumigatus* | -- | KT852626 | South(Fuzhou) |
| SHTJ0064 | *Aspergillus fumigatus* | -- | KT852627 | South(Fuzhou) |
| SHTJ0065 | *Aspergillus fumigatus* | sputum | KT932354 | South(Fuzhou) |
| SHTJ0066 | *Aspergillus fumigatus* | sputum | KT852628 | North(Hebei) |
| SHTJ0067 | *Aspergillus fumigatus* | sputum | KT852629 | North(Hebei) |
| SHTJ0068 | *Aspergillus fumigatus* | sputum | KT852630 | North(Hebei) |
| SHTJ0069 | *Aspergillus fumigatus* | sputum | KT852631 | North(Hebei) |
| SHTJ0070 | *Aspergillus fumigatus* | sputum | KT852632 | North(Hebei) |
| SHTJ0071 | *Aspergillus fumigatus* | sputum | KT852633 | North(Hebei) |
| SHTJ0072 | *Aspergillus fumigatus* | sputum | KT852634 | North(Hebei) |
| SHTJ0073 | *Aspergillus fumigatus* | sputum | KT852635 | North(Hebei) |
| SHTJ0074 | *Aspergillus fumigatus* | sputum | KT852636 | North(Hebei) |
| SHTJ0075 | *Aspergillus fumigatus* | sputum | KT852637 | North(Hebei) |
| SHTJ0076 | *Aspergillus fumigatus* | sputum | KT852638 | North(Hebei) |
| SHTJ0077 | *Aspergillus fumigatus* | sputum | KT852639 | North(Hebei) |
| SHTJ0078 | *Aspergillus fumigatus* | sputum | KT852640 | North(Hebei) |
| SHTJ0079 | *Aspergillus fumigatus* | sputum | KT852641 | North(Hebei) |
| SHTJ0080 | *Aspergillus fumigatus* | sputum | KT852642 | North(Hebei) |
| SHTJ0081 | *Aspergillus fumigatus* | sputum | KT852643 | North(Hebei) |
| SHTJ0082 | *Aspergillus fumigatus* | sputum | KT852644 | North(Hebei) |
| SHTJ0083 | *Aspergillus fumigatus* | sputum | KT852645 | North(Hebei) |
| SHTJ0084 | *Aspergillus fumigatus* | sputum | KT852646 | North(Hebei) |
| SHTJ0085 | *Aspergillus fumigatus* | sputum | KT852647 | North(Hebei) |
| SHTJ0086 | *Aspergillus fumigatus* | sputum | KT852648 | North(Hebei) |
| SHTJ0087 | *Aspergillus fumigatus* | sputum | KT852649 | North(Hebei) |
| SHTJ0088 | *Aspergillus fumigatus* | sputum | KT852650 | North(Hebei) |
| SHTJ0089 | *Aspergillus fumigatus* | sputum | KT852651 | North(Hebei) |
| SHTJ0090 | *Aspergillus fumigatus* | sputum | KT852652 | North(Hebei) |
| SHTJ0091 | *Aspergillus fumigatus* | sputum | KT852653 | North(Hebei) |
| SHTJ0092 | *Aspergillus fumigatus* | sputum | KT852654 | North(Hebei) |
| SHTJ0093 | *Aspergillus fumigatus* | sputum | KT852655 | North(Hebei) |
| SHTJ0094 | *Aspergillus fumigatus* | sputum | KT852656 | North(Hebei) |
| SHTJ0095 | *Aspergillus fumigatus* | sputum | KT852657 | North(Hebei) |
| SHTJ0096 | *Aspergillus fumigatus* | sputum | KT852658 | North(Hebei) |
| SHTJ0097 | *Aspergillus fumigatus* | sputum | KT852659 | North(Hebei) |
| SHTJ0098 | *Aspergillus fumigatus* | sputum | KT852660 | North(Hebei) |
| SHTJ0099 | *Aspergillus fumigatus* | sputum | KT852661 | North(Hebei) |
| SHTJ0100 | *Aspergillus fumigatus* | sputum | KT852662 | North(Hebei) |
| SHTJ0101 | *Aspergillus fumigatus* | sputum | KT852663 | East South(Shanghai) |
| SHTJ0102 | *Aspergillus fumigatus* | sputum | KT852664 | East South(Shanghai) |
| SHTJ0103 | *Aspergillus fumigatus* | sputum | KT852665 | East South(Shanghai) |
| SHTJ0104 | *Aspergillus fumigatus* | sputum | KT852666 | East South(Shanghai) |
| SHTJ0105 | *Aspergillus fumigatus* | sputum | KT852667 | East South(Shanghai) |
| SHTJ0106 | *Aspergillus fumigatus* | sputum | KT852668 | East South(Shanghai) |
| SHTJ0107 | *Aspergillus fumigatus* | sputum | KT852669 | East South(Shanghai) |
| SHTJ0108 | *Aspergillus fumigatus* | sputum | KT852670 | East South(Shanghai) |
| SHTJ0109 | *Aspergillus fumigatus* | sputum | KT852671 | East South(Shanghai) |
| SHTJ0110 | *Aspergillus fumigatus* | sputum | KT852672 | East South(Shanghai) |
| SHTJ0111 | *Aspergillus fumigatus* | sputum | KT852673 | East South(Shanghai) |
| SHTJ0112 | *Aspergillus fumigatus* | sputum | KT852674 | East South(Shanghai) |
| SHTJ0113 | *Aspergillus fumigatus* | sputum | KT852675 | East South(Shanghai) |
| SHTJ0114 | *Aspergillus fumigatus* | sputum | KT852676 | East South(Shanghai) |
| SHTJ0115 | *Aspergillus fumigatus* | throat swab | KT852677 | East South(Shanghai) |
| SHTJ0116 | *Aspergillus fumigatus* | sputum | KT852678 | East South(Shanghai) |
| SHTJ0117 | *Aspergillus fumigatus* | sputum | KT852679 | East South(Shanghai) |
| SHTJ0119 | *Aspergillus fumigatus* | sputum | KT852680 | East South(Shanghai) |
| SHTJ0121 | *Aspergillus fumigatus* | sputum | KT852681 | East South(Shanghai) |
| SHTJ0122 | *Aspergillus fumigatus* | sputum | KT852682 | East South(Shanghai) |
| SHTJ0123 | *Aspergillus fumigatus* | sputum | KT852683 | East South(Shanghai) |
| CZLIAOWQ2015AF | *Aspergillus fumigatus* | sputum | KU714964 | East South(Shanghai) |
| SHTJ0129 | *Aspergillus fumigatus* | sputum | KT852687 | East South(Nanjing) |
| SHTJ0130 | *Aspergillus fumigatus* | sputum | KT852688 | East South(Nanjing) |
| SHTJ0131 | *Aspergillus fumigatus* | sputum | KT852689 | East South(Nanjing) |
| SHTJ0132 | *Aspergillus fumigatus* | sputum | KT852690 | East South(Nanjing) |
| SHTJ0133 | *Aspergillus fumigatus* | sputum | KT852691 | East South(Nanjing) |
| SHTJ0134 | *Aspergillus fumigatus* | sputum | KT852692 | East South(Nanjing) |
| SHTJ0135 | *Aspergillus fumigatus* | sputum | KT852693 | East South(Nanjing) |
| SHTJ0136 | *Aspergillus fumigatus* | BALF | KT852694 | East South(Nanjing) |
| SHTJ0137 | *Aspergillus fumigatus* | sputum | KT852695 | East South(Nanjing) |
| SHTJ0138 | *Aspergillus fumigatus* | sputum | KT852696 | East South(Nanjing) |
| SHTJ0139 | *Aspergillus fumigatus* | sputum | KT852697 | East South(Nanjing) |
| SHTJ0140 | *Aspergillus fumigatus* | sputum | KT932355 | East South(Nanjing) |
| SHTJ0141 | *Aspergillus fumigatus* | sputum | KT852698 | East South(Nanjing) |
| SHTJ0142 | *Aspergillus fumigatus* | sputum | KT852699 | East South(Nanjing) |
| SHTJ0143 | *Aspergillus fumigatus* | sputum | KT852700 | East South(Nanjing) |
| SHTJ0125 | *Aspergillus fumigatus* | sputum | KT852684 | West(Urumqi) |
| SHTJ0126 | *Aspergillus fumigatus* | sputum | KT852685 | West(Urumqi) |
| SHTJ0128 | *Aspergillus fumigatus* | sputum | KT852686 | West(Urumqi) |
| XYZ10106 | *Aspergillus fumigatus* | sputum | KU558729 | West(Urumqi) |
| XYZ10107 | *Aspergillus fumigatus* | sputum | KU714947 | West(Urumqi) |
| XYZ10112 | *Aspergillus fumigatus* | sputum | KU714948 | West(Urumqi) |
| XYZ10116 | *Aspergillus fumigatus* | sputum | KU714949 | West(Urumqi) |
| XYZ10117 | *Aspergillus fumigatus* | sputum | KU714950 | West(Urumqi) |
| XYZ10118 | *Aspergillus fumigatus* | sputum | KU714951 | West(Urumqi) |
| XYZ10119 | *Aspergillus fumigatus* | sputum | KU714952 | West(Urumqi) |
| XYZ10120 | *Aspergillus fumigatus* | sputum | KU714953 | West(Urumqi) |
| XYZ10121 | *Aspergillus fumigatus* | sputum | KU714954 | West(Urumqi) |
| XYZ10124 | *Aspergillus fumigatus* | sputum | KU558730 | West(Urumqi) |
| XYZ10134 | *Aspergillus fumigatus* | sputum | KU714955 | West(Urumqi) |
| XYZ10135 | *Aspergillus fumigatus* | sputum | KU714956 | West(Urumqi) |
| XYZ10138 | *Aspergillus fumigatus* | sputum | KU714957 | West(Urumqi) |
| XYZ10144 | *Aspergillus fumigatus* | sputum | KU714958 | West(Urumqi) |
| XYZ10148 | *Aspergillus fumigatus* | sputum | KU714959 | West(Urumqi) |
| XYZ10151 | *Aspergillus fumigatus* | sputum | KU714960 | West(Urumqi) |
| XYZ10154 | *Aspergillus fumigatus* | sputum | KU714961 | West(Urumqi) |
| XYZ10155 | *Aspergillus fumigatus* | sputum | KU714962 | West(Urumqi) |
| XYZ10156 | *Aspergillus fumigatus* | sputum | KU714963 | West(Urumqi) |

-- : source unknown.

BALF: bronchial alveolar lavage fluid. CZLIAOWQ2015AF
