# Supplementary material for: Triazole phenotypes and genotypic characterization of clinical Aspergillus fumigatus isolates in China
Source: Emerg Microbes Infect. 2017 Dec 6;6(12):e109–. doi: 10.1038/emi.2017.97 (PMC5750463; doi:10.1038/emi.2017.97)
Supplement: Supplementary Table S2 [file emi201797x2.docx]

**Supplementary Table S2** Primers and annealing temperatures used in this study

| Primers name | Annealing temperatures | Primers |
| --- | --- | --- |
| Promoter region | 53 ℃ | Fp 5’–GGA GAT ACT ATG GCT TTC AT–3’  Rp 5’–GTA TGC TGG AAC TAC ACC T–3’ |
| cyp51A primer 1 | 57 ℃ | F1 5’–ATG GTG CCG ATG CTA TGG–3’  R1 5’–TAC TGG AGC GGA GGA AGA–3’ |
| cyp51A primer 2 | 53 ℃ | F2 5’–GAT AAA GAG ATT GCG CAC AT–3’  R2 5’–ACA GTC TCA CTT GGA TGT G–3’ |

Abbreviations: F, Forward primer; R, Reverse primer.
